# Total Intravenous Anaesthesia and an Opioid-Sparing Strategy for an Extended Left Hemihepatectomy with Total Caudate Resection, Total Hepatic Vascular Exclusion, and Venovenous Bypass: An Integrative Anaesthesia Approach

**DOI:** 10.7759/cureus.91936

**Published:** 2025-09-09

**Authors:** Sing Ying Pang, Jan Ngian, Selene Tan

**Affiliations:** 1 Department of Anaesthesiology, Singapore General Hospital, Singapore, SGP

**Keywords:** major hepatectomy, multimodal analgesia, sustainable anaesthesia, total hepatic vascular exclusion, total intravenous anaesthesia

## Abstract

We present a case of a 72-year-old man who underwent an open extended left hemihepatectomy and total caudate lobe resection with total hepatic vascular exclusion (THVE) for a large hepatocellular carcinoma (HCC). Anaesthetic management included the use of intrathecal opioids and total intravenous anaesthesia (TIVA) with propofol and remifentanil. Due to tumour adherence to the right hepatic vein-inferior vena cava junction, venovenous (VV) bypass and cold in situ perfusion were required. This case illustrates neurocognitive protection principles, electroencephalogram (EEG)-guided depth of anaesthesia monitoring, multimodal opioid-sparing analgesic strategies, enhanced recovery principles, and sustainable, low-emission TIVA anaesthetic maintenance, exemplifying an integrative approach in high-complexity liver surgery.

## Introduction

Major hepatic resections are among the most technically and physiologically demanding surgical procedures. Risks include but are not limited to massive bleeding, profound haemodynamic instability, and postoperative liver failure, to name a few. Anaesthetic goals include providing optimal surgical conditions, physiologic homeostasis, haemostatic management, amelioration of the surgical stress response with multimodal opioid-sparing analgesia (MMA) and postoperative recovery optimisation [1]. Additionally, the role of the anaesthetist has expanded to encompass considerations of neurocognitive protection, enhanced recovery after surgery (ERAS), environmental sustainability and resource stewardship [2, 3]. Combining total intravenous anaesthesia (TIVA), the technique whereby general anaesthesia is maintained entirely using intravenous agents, and MMA, a pain management strategy incorporating multiple techniques with different mechanisms of action to provide pain control with an improved side effect profile, may confer smoother anaesthetic emergence, reduce the risk of postoperative nausea and vomiting, facilitate ERAS and reduce the carbon footprint of a long, complex surgery. This report presents the anaesthetic management of a patient undergoing extended left hemihepatectomy with total caudate lobe resection, total hepatic vascular exclusion (THVE), and venovenous (VV) bypass. THVE, involving complete cessation of hepatic blood inflow and outflow, presents unique anaesthetic challenges, including altered drug pharmacokinetics and profound haemodynamic changes, while VV bypass, reserved for the most complex cases where THVE alone is not haemodynamically tolerated, adds further physiological complexity requiring meticulous monitoring and management. This case demonstrates the feasibility of a high-complexity integrative approach in such demanding surgical circumstances.

## Case presentation

**Figure 1:**
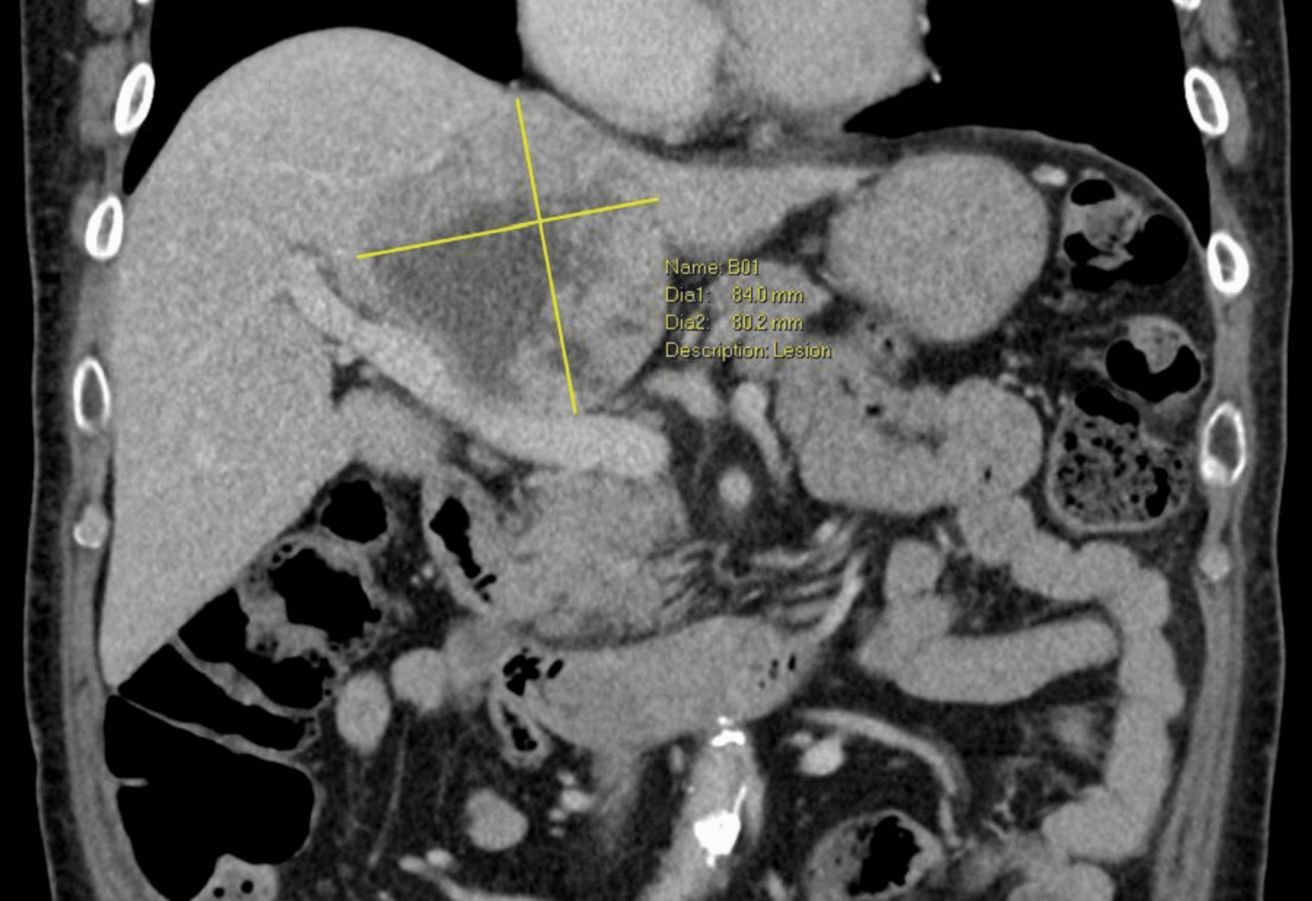
Coronal computed tomography (CT) venous phase demonstrating a large, centrally located tumour close to the portal vein.

**Figure 2:**
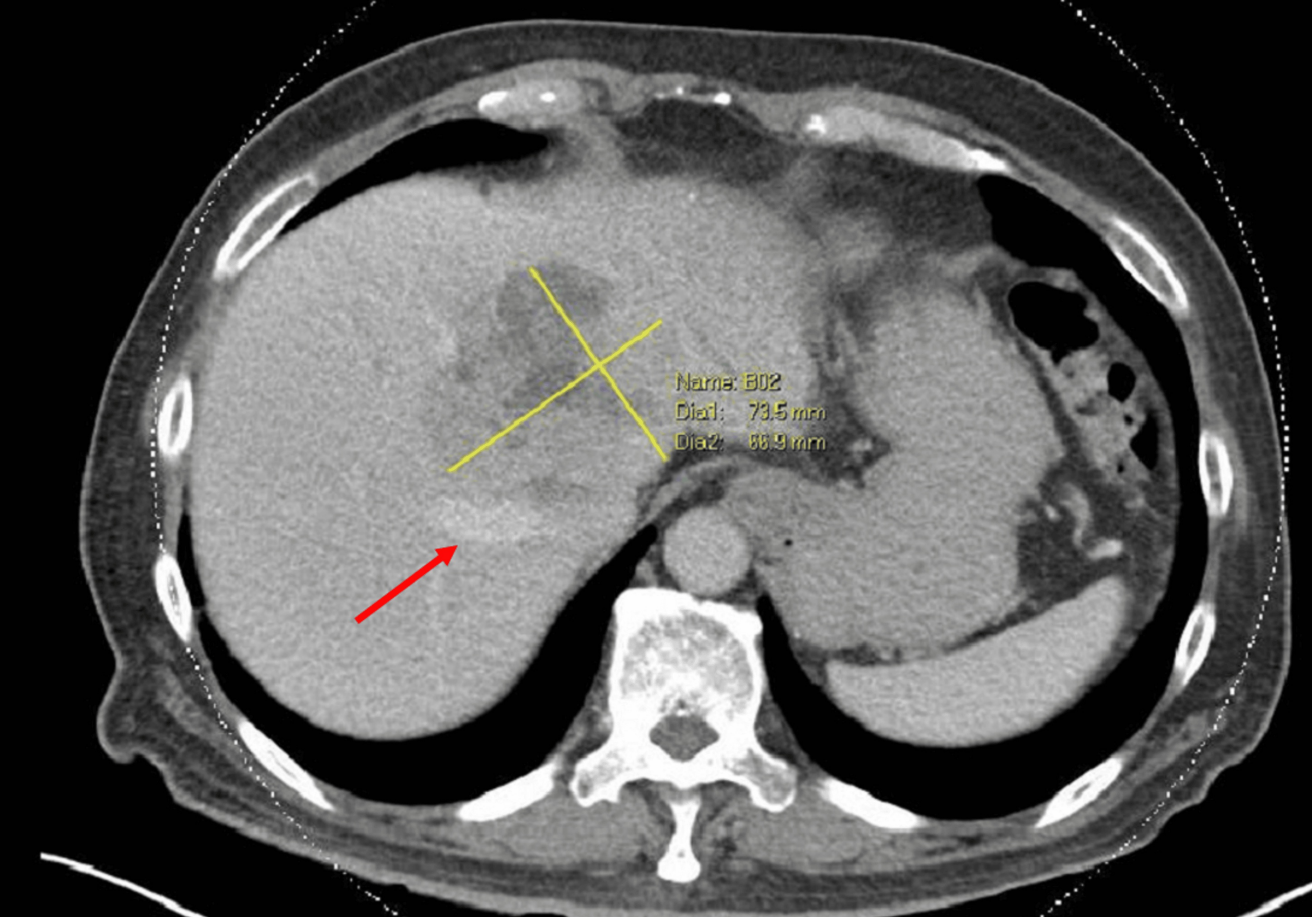
Axial CT venous phase again demonstrating a large, centrally located lesion abutting the middle hepatic vein (red arrow).

A 72-year-old patient presented for open extended left hemihepatectomy and total caudate lobe resection for an 8 cm hepatocellular carcinoma (HCC). He had ocular myasthenia gravis on mycophenolate, mofetil, and pyridostigmine. An incidental finding of abnormal liver function tests was noted. He was sent for ultrasound and computed tomography (CT) imaging of the liver, which revealed an HCC abutting the right hepatic vein (RHV) and inferior vena cava (IVC), displacing the middle hepatic vein (MHV) and effacing the left hepatic vein (LHV). The portal vein appeared preserved (Figures 1, 2). He received a course of selective internal radiation therapy (SIRT) with yttrium-90 before proceeding with surgery. 

The patient was admitted on the morning of surgery under an ERAS pathway. Intrathecal morphine 150 mcg and fentanyl 30 mcg were administered prior to general anaesthesia with endotracheal intubation and TIVA (target-controlled infusions of propofol 2-3 mcg/ml, Schnider model, effect site; and remifentanil 0.1-2.5 ng/ml, Minto model, effect site). Neuromuscular blockade was maintained with a rocuronium infusion of 0.35 mg/kg/h. Vascular access included a right radial 20-gauge (G) arterial line, rapid volume and vasopressor infusion lines (a left internal jugular vein quadruple-lumen 8.5 French (Fr) central venous catheter (CVC) and 8.5 Fr pulmonary artery catheter introducer sheath), and pre-placement of lines for potential VV bypass, a right internal jugular vein 16G intravenous (IV) cannula and right femoral vein single-lumen CVC. 

A Masimo SedLine® Brain Monitoring Electroencephalogram (EEG) monitor (Masimo Corporation, Irvine, CA) was used to titrate depth of anaesthesia, targeting Patient State Index (PSI) readings between 30 and 50, bilateral spectral edge frequencies (SEF95) of <12 Hz, and characteristic theta-delta EEG waveform dominance while avoiding burst suppression. IV Plasma-Lyte A solution was administered at a low rate of 1-2 ml/kg/hr in order to minimise hepatic venous pressures for liver resection. Central venous pressure (CVP) was kept below 5 mmHg, and mean arterial pressure (MAP) was maintained above 65 mmHg. 

**Figure 3:**
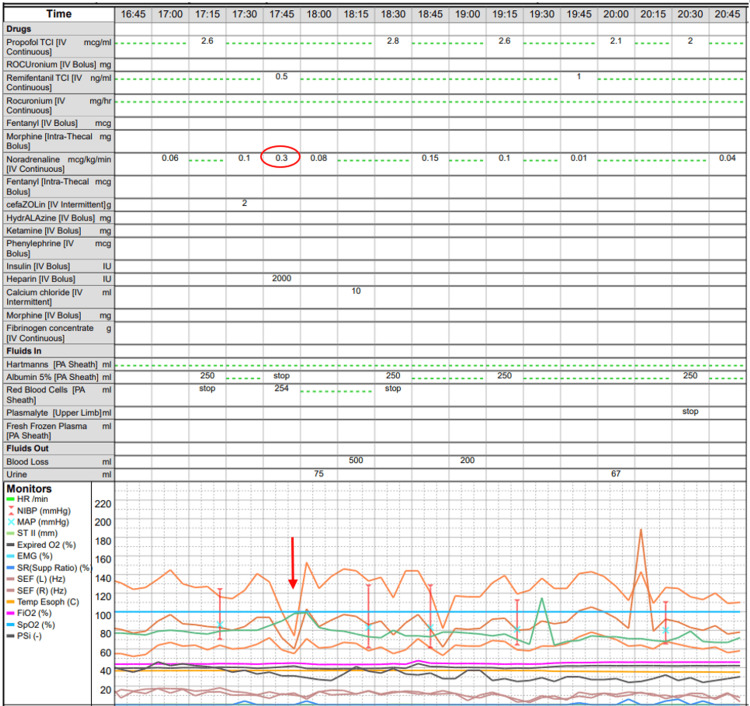
Anaesthetic chart demonstrating intraoperative profound hypotension (red arrow) despite noradrenaline at 0.03mcg/kg/min (red circle) demonstrating failed attempt at THVE prior to VV bypass. Orange lines denote intra-arterial systolic, mean and diastolic blood pressures. THVE: total hepatic vascular exclusion; VV: venovenous

Intraoperatively, the tumour was found to be densely adherent to the RHV-IVC junction and IVC. THVE without VV bypass was attempted but not tolerated despite vasopressor support with noradrenaline up to 0.3 mcg/kg/min (Figure 3).

VV bypass was initiated with outflow via the right femoral vein and inflow via the right internal jugular vein. Cold in situ perfusion of the liver was performed for 90 min during the total VV bypass time of 90 min, during which tumour resection and IVC reconstruction were completed. Six cycles of inflow occlusion (Pringle manoeuvre) were applied. Total blood loss was 3000 ml. Blood product administration included 1000 ml of packed red cells, 530 ml of fresh frozen plasma, 2.5 L of 5% albumin and 1.5 L of Plasma-Lyte A. Additionally, 2g of fibrinogen concentrate was administered with rotational thromboelastometry (ROTEM) guidance (fibrin-specific ROTEM (FIBTEM) A5 of 6mm). Arterial blood gas analysis showed a pH of 7.27 to 7.28 and a base excess of -5 to -8 post-reperfusion of the liver. Arterial blood gases and lactate readings normalised by postoperative day (POD) 1 (Table 1).

**Table 1:** Arterial blood gas analysis: post-reperfusion and postoperative day 1 PaCO_2_: partial pressure of carbon dioxide; PaO_2_: partial pressure of oxygen

Test	35 minutes post reperfusion	86 minutes post reperfusion	Postoperative day 1	Reference values
pH	7.28	7.27	7.34	7.35-7.45
PaCO_2_	46.8	43.3	40.8	35-45 mmHg
PaO_2_	214	226	216	80-105 mmHg
Base excess	-5	-7	-4.5	-2 - +3 mmol/L
Bicarbonate	21.9	20	21.4	22-26 mmol/L
Lactate	Not available	2.5	2.2	0.5-2.2 mmol/L

IV ketamine 0.4 mg/kg was administered over the course of surgery. IV morphine 0.05 mg/kg was administered at the end of surgery. The patient was transferred intubated to the surgical intensive care unit (SICU) but extubated the next day and discharged from SICU on POD 1. Noradrenaline infusion was weaned off by the time of transfer to the SICU.

Postoperative recovery was uneventful. Pain scores ranged from 0 to two at rest and on movement. Opioid consumption was minimal, at a total of 100 mcg of fentanyl delivered via a patient-controlled analgesia pump over PODs 1 to 3. The patient emerged from anaesthesia in an alert and calm state. There was no postoperative pruritus, nausea, vomiting, delirium, or confusion. He was discharged from the hospital on POD 7. 

## Discussion

This case highlights the layered demands of a technically challenging, complex liver surgery and the successful implementation of an integrative anaesthetic technique that addressed both physiological and broader systems-level goals. The use of THVE is relatively uncommon and is indicated for resection of tumours involving or adjacent to the vena cava and/or confluence of the hepatic veins [4]. The use of TIVA guided by EEG monitoring allowed precise titration of anaesthetic depth while avoiding burst suppression, minimising neurocognitive risk. Despite a major blood loss of about 3000 ml during the surgery, propofol requirements remained relatively stable throughout surgery. MAP was also maintained above 65 mmHg with goal-directed fluid and blood replacement therapy and vasopressors as required. 

Broader goals include facilitation of the ERAS protocols to increase efficiency and reduce costs in the system [5], with strategies for preoperative optimisation and a multimodal pain strategy to minimise risk of postoperative cognitive dysfunction (POCD) and postoperative nausea and vomiting (PONV) in our increasingly elderly patients. 

Beyond the care of a single patient, the recent evolution of the practice of anaesthesia has also included a shift in focus to sustainable practices driven by environmental concerns. The carbon footprint of inhaled anaesthetics has been an increasing concern for discussion in recent years [3], and in general, the use of TIVA is also in line with a more environmentally conscious approach [6-8].

ERAS has been shown to decrease complication rates and length of stay, and hence costs. It begins in the preoperative phase, where high-risk patients have been shown to benefit from four to six weeks of prehabilitation and nutritional optimisation [5]. Our patient was relatively well to begin with and did not require prehabilitation. On the day of surgery, long-acting anxiolytics were avoided, and a multimodal pain technique with intrathecal opioids and the use of ultra-short-acting agents such as remifentanil, ketamine, and minimal morphine was used. As can be seen, the postoperative pain scores and opioid consumption demonstrated satisfactory integration of TIVA with multimodal analgesia. 

Propofol is purported to reduce CVP by inducing vasodilatation, resulting in less hepatic venous distension and hence reducing blood loss [9]. However, haemorrhage is inevitable in surgeries as major as these. Blood loss from central and extended hepatectomies can range from 400 ml to about 2200 ml, depending on complexity [10], and in comparison, this case surpassed that. Propofol undergoes extensive hepatic metabolism with a blood extraction ratio of about 90% [11]. During THVE and VV bypass, propofol pharmacokinetics would have been dramatically altered, essentially eliminating hepatic clearance during the 90-minute bypass period and leading to uncertain drug plasma concentration. Sedline EEG monitoring allowed us to titrate depth of anaesthesia accurately, which was additionally important given this patient’s history of myasthenia gravis. 

## Conclusions

This case uniquely demonstrates that principles of sustainability, such as TIVA and ERAS can be successfully and safely integrated even in major abdominal surgery such as major liver resections requiring VV bypass, and if widely applied, may have a deep impact on healthcare-related carbon emissions. The ideal is to minimise environmental impact and yet preserve safety and efficacy of treatment for patients, striking a balance between maintaining quality care and reduced resource use. As increasingly elderly patients come for such surgery, POCD is becoming a significant concern alongside PONV and effective pain management strategies. More studies are required to confirm the feasibility of such practices.
